# *Brachyspira hyodysenteriae* Infection Reduces Digestive Function but Not Intestinal Integrity in Growing Pigs While Disease Onset Can Be Mitigated by Reducing Insoluble Fiber

**DOI:** 10.3389/fvets.2020.587926

**Published:** 2020-10-26

**Authors:** Emma T. Helm, Susanne J. Lin, Nicholas K. Gabler, Eric R. Burrough

**Affiliations:** ^1^Department of Animal Science, Iowa State University, Ames, IA, United States; ^2^Department of Veterinary Pathology, Iowa State University, Ames, IA, United States; ^3^Department of Veterinary Diagnostic and Production Animal Medicine, Iowa State University, Ames, IA, United States

**Keywords:** pig, *Brachyspira hyodysenteriae*, intestinal integrity, digestibility, nutrition, fermentable fiber, insoluble fiber

## Abstract

Swine dysentery (**SD**) induced by *Brachyspira hyodysenteriae* manifests as mucohemorrhagic diarrhea in pigs, but little is known about the changes that occur to the gastrointestinal tract during this disease. It is thought that dietary fibers alter disease pathogenesis, although the mechanisms of action are unclear. Thus, the objectives of this study were to characterize intestinal integrity, metabolism, and function in pigs during SD and determine if replacing insoluble fiber with fermentable fibers mitigates disease. Thirty-six *B. hyodysenteriae*-negative gilts [24.3 ± 3.6 kg body weight (BW)] were assigned to one of three treatment groups: (1) *B. hyodysenteriae* negative, control diet (**NC**); (2) *B. hyodysenteriae* challenged, control diet (PC); and (3) *B. hyodysenteriae* challenged, highly fermentable fiber diet (RS). The NC and PC pigs were fed the same control diet, containing 20% corn distillers dried grains with solubles (DDGS). The RS pigs were fed a diet formulated with 5% sugar beet pulp and 5% resistant potato starch. On days post inoculation (dpi) 0 and 1, pigs were inoculated with *B. hyodysenteriae* or sham. Pigs were euthanized for sample collection after onset of SD. The challenge had high morbidity, with 100% of PC and 75% of RS pigs developing clinical SD. The timing of onset of clinical SD differed due to treatment, with RS pigs having a delayed onset (dpi 9) of clinical SD compared with dpi 7 for PC pigs. Colon transepithelial resistance was increased and macromolecule permeability was reduced in PC pigs compared with NC pigs (*P* < 0.01). Minimal changes in ileal permeability, mitochondrial function, or volatile fatty acids (VFAs) were observed. Total VFA concentrations were lower in the colon and cecum in both PC and RS pigs compared to NC pigs (both *P* < 0.05), but iso-acids were higher (both *P* < 0.05). Total tract digestibility of dry matter (DM), organic matter (OM), nitrogen (N), and gross energy (GE) was lower in PC pigs compared with both NC and RS pigs (both *P* < 0.001). These data indicate that SD reduces digestive function but does not reduce *ex vivo* intestinal integrity. Further, replacement of insoluble fiber with highly fermentable fibers mitigated and delayed the onset of SD.

## Introduction

Swine dysentery (**SD**) is an economically significant disease of growing pigs characterized by mucohemorragic diarrhea, or watery feces containing large amounts of mucus and flecks of blood (1). Pigs clinically affected with SD become anorexic and dehydrated, resulting in rapid weight loss (2). The causative agents of SD include strongly hemolytic *Brachyspira* spp., such as *Brachyspira hyodysenteriae* and *Brachyspira hampsonii* (2). Upon association with the colonic epithelium, *B. hyodysenteriae* causes marked inflammation, hemorrhage, superficial epithelial necrosis, and excessive production of mucus (3, 4). Further, the mucus layer becomes thickened and disorganized, potentially providing *B. hyodysenteriae*, a favorable niche for binding and proliferation (4).

Diarrhea during *B. hyodysenteriae* infection is driven by complete abolition of water absorption (5) and reduces apparent total tract digestibility of nutrients and energy, which contributes to attenuated growth performance (6). Additionally, inflammation at the colonic epithelium may alter intestinal metabolism to favor glycolysis, exacerbating dysbiosis (7). Few studies have evaluated intestinal barrier permeability, digestive function, and intestinal metabolism in *B. hyodysenteriae*-challenged pigs. Further, as *B. hyodysenteriae* is a large intestinal pathogen, investigations have been primarily limited to the cecum and colon (5, 8, 9). However, as the small intestine is the primary site of nutrient digestion and absorption (10), understanding if *B. hyodysenteriae* infection alters small intestinal integrity and function is highly relevant and applicable to developing mitigation strategies.

Although there is discord in the literature, dietary fiber manipulation is known to affect the clinical presentation of SD upon experimental infection (11, 12). Most of these studies examining dietary influences on SD development have utilized base ingredients such as white rice, wheat, barley, and triticale, which are more prominently used in European and Australasian pig production regions. In the US, recent research has shown that high dietary inclusion of corn distillers dried grains with solubles (DDGS), an ingredient high in insoluble fiber, increased SD disease severity in growing pigs (13). It is speculated that by limiting fermentation in the hindgut, SD severity decreases (11, 14), but certain highly fermentable fiber types may increase or decrease disease depending on fiber type and the base diet utilized (11, 12, 15). Inclusion or exclusion of certain dietary fibers may alter disease pathogenesis by changing the physiochemical environment of the large intestine or by modulating microbial communities and microbial fermentation products to either increase or decrease the ability of *B. hyodysenteriae* to associate with the intestinal epithelium and cause disease. However, these mechanisms of action have not been fully explored.

Thus, the objective of the study herein was to characterize changes to small and large intestinal integrity, metabolism, and function in grower pigs experiencing SD after *B. hyodysenteriae* infection. Further, we aimed to determine if replacement of dietary insoluble fiber with highly fermentable fibers would mitigate the development and severity of SD.

## Materials and Methods

All animal procedures were approved by the Iowa State University Institutional Animal Care and Use Committee (IACUC protocol #19-170) and adhered to the guidelines for ethical and humane use of animals for research.

### Animals, Diets, and Experimental Design

A total of 36 gilts from a herd with no history of SD [24.3 ± 3.6 kg body weight (BW); Camborough (1050) X 337 Hendersonville, TN] were randomly selected for this experiment. Pigs were individually confirmed negative for *B. hyodysenteriae via* selective culture of fecal swabs, allocated to individual pens, and assigned treatment groups. Pigs were allotted across three treatment groups: (1) *B. hyodysenteriae* negative, fed the control diet (**NC**, *n* = 12); (2) *B. hyodysenteriae* challenged, fed the control diet (PC, *n* = 12); and (3) *B. hyodysenteriae* challenged, fed a highly fermentable fiber diet (RS, *n* = 12). Pigs were housed across two rooms in the same barn, in which NC pigs were housed in a separate room to prevent pathogen spread. The two rooms had identical pen sizes, feeders, flooring, heating, cooling, and water supply, but separate manure pits.

The NC and PC pigs were fed the same corn-soybean meal control diet, formulated with 20% corn DDGS. The RS pigs were fed a corn-soybean meal diet formulated with 5% sugar beet pulp and 5% resistant potato starch added at the expense of DDGS. Diets were *ad libitum* fed throughout the experiment and met or exceeded all National Research Council (16) requirements and contained no antibiotics (Table 1). Both diets were formulated to be isocaloric (3,300 kcal/kg metabolizable energy) and isonitrogenous (18% crude protein) and to have the same level of standardized ileal digestible lysine (0.98%). Pigs were started on dietary treatments 3 weeks prior to initiation of the *B. hyodysenteriae* challenge to allow for microbial adaptation.

**Table 1:** Diet composition, as fed.

**Ingredient**	**Control**	**RS**
Corn	57.28	54.80
Soybean meal	18.40	20.60
Corn DDGS^a^	20.00	10.00
Soybean oil	1.00	1.27
Salt	0.35	0.35
Monocalcium phosphate, 21%	0.63	0.78
Limestone	1.24	1.03
L-lysine HCl	0.36	0.34
L-threonine	0.04	0.07
DL-methionine	–	0.06
Vitamin premix^b^	0.15	0.15
Trace mineral premix^c^	0.15	0.15
TiO_2_	0.40	0.40
Resistant potato starch	–	5.00
Sugar beet pulp	–	5.00
*Calculated composition*		
Metabolizable energy, kcal/kg	3,341	3,300
Crude protein, %	19.36	17.97
SID lysine^d^, %	0.98	0.98
*Analyzed composition*		
Dry matter, %	88.50	88.78
Crude protein, %	18.01	17.62
Gross energy, kcal/kg	3,817	3,857
Acid detergent fiber, %	4.07	4.34
Neutral detergent fiber, %	11.47	9.93
Insoluble dietary fiber, %	12.52	10.78
Total dietary fiber, %	12.95	11.04
Resistant starch, %	2.61	6.31
Total starch, %	34.99	37.82

a *DDGS, corn distiller's dried grains with solubles*.

b *Provided per kilogram of diet: 6,125 IU vitamin A, 700 IU vitamin D_3_, 50 IU vitamin E, 30 mg vitamin K, 0.05 mg vitamin B_12_, 11 mg riboflavin, 56 mg niacin, and 27 mg pantothenic acid*.

c *Provided per kilogram of diet: 22 mg Cu (as CuSO_4_), 220 mg Fe (as FeSO_4_), 0.4 mg I [as Ca(IO_3_)_2_], 52 mg Mn (as MnSO_4_), 220 mg Zn (as ZnSO_4_), and 0.4 mg Se (as Na_2_SeO_3_)*.

a *SID, standardized ileal digestibility*.

On days post inoculation (dpi) 0 and 1, pigs were inoculated with *B. hyodysenteriae* or sham. The *B. hyodysenteriae* (B204) strain used was originally recovered from a clinical case of SD in 1972 and was obtained from the culture collection at the Iowa State University Veterinary Diagnostic Laboratory (ISU VDL). Inoculum was prepped according to Burrough et al. (17). Briefly, the isolate was thawed, plated onto several tryptic soy agar plates, and anaerobically incubated. Inoculum was prepared by removing the agar, placing it into a bag, and homogenizing the agar by hand until a slurry of uniform consistency was achieved. A small sample of each day's slurry was retained for determination of inoculum concentration by standard plate counting. The *B. hyodysenteriae*-challenged pigs received two doses of inoculum (60 ml/dose, containing approximately 3.4 × 10^6^ and 3.9 × 10^6^ colony-forming units/ml) administered as an agar slurry *via* gastric gavage as previously described (17). The sham-inoculated pigs received two 60-ml doses of agar slurry *via* gastric gavage. Inoculations were performed 24 h apart with each administration preceded by a 12–18-h fast. Throughout the study period, fecal consistency was evaluated each morning by the same individual who was blinded to dietary treatment. Fecal consistency was scored based on the following system: 0 if normal, 1 if soft but formed, 2 if semisolid, and 3 if liquid to watery. An additional 0.5 point was added each for the presence of discernible mucus and/or blood for a total maximum score of 4. Pigs were considered to have clinical SD upon scoring a 4 (liquid to watery diarrhea with presence of blood and mucus). At dpi −14, 0, 4, 8, and 12 and at necropsy, individual pig BW and individual pig feed disappearance were recorded to calculate performance parameters of average daily gain (ADG), average daily feed intake (ADFI), and feed efficiency [gain:feed (G:F)].

Pigs were euthanized within 72 h of the initial observation of clinical SD or at the end of the study on dpi 16 (between dpi 10 and 16). Pigs were euthanized in repetitions of 6–8 pigs per repetition, with at least two NC pigs included in each repetition. Pigs were euthanized by captive bolt followed by exsanguination, and tissues were collected for analysis. Sections from the ileum, collected 30 cm orad from the ileal-cecal junction, and the apex of the spiral colon were placed in continuously aerated bottles containing Krebs buffer (25 mM NaHCO_3_, 120 mM NaCl, 1 mM MgSO_4_, 6.3 mM KCl, 2 mM CaCl_2_, and 0.32 mM NaH_2_PO_4_) and for transport to the laboratory for analysis. Adjacent sections of the ileum and spiral colon were rinsed in Krebs buffer, frozen in liquid nitrogen, and then stored at −80°C until analysis. An additional section of the spiral colon apex was aseptically collected and placed on ice until selective anaerobic culture for *B. hyodysenteriae* using routine methodology at the ISU VDL.

Contents from the ileum, cecum, and spiral colon were collected into 50-ml conical tubes. A portable pH probe (Thermo Fisher Scientific, Waltham, MA) was utilized to measure pH of the contents, after which contents were frozen and stored at −80°C until further analysis.

### *Ex vivo* Barrier Function and Integrity

Freshly isolated ileum and colon sections were mounted on modified Using chambers (Physiologic Instruments Inc., San Diego, CA) within ~1 to 1.5 h of euthanasia. Upon receipt to the laboratory, the serosal layer of intestinal tissues was removed, lumens were opened, and the mucosa was visually assessed for intact and undisturbed adhered mucus and epithelial layers. Explants were selected from tissue regions that remained undisturbed. Modified Using chambers were assembled, and electrophysiological and fluorescein isothiocyanate-dextran 4 kDa (FD4) macromolecule permeability measurements were collected as previously described (18). Estimates of nutrient transport for ileal samples were calculated as the change in current (μA) after glucose and glutamine addition. A fluorescent plate reader (Cytation 5 Hybrid Multi-Mode Reader, BioTek Instruments Inc., Winooski, VT) was used to determine mucosal to serosal flux changes in relative fluorescence of FD4 in the serosal samples from 0 to 60 min after FD4 addition at 485 and 520 nm excitation and emission wavelengths, respectively. Apparent permeability coefficients for FD4 flux were calculated as described by Pearce et al. (19).

### Mitochondrial Isolation, Reactive Oxygen Species Production, and Oxygen Consumption

Efficient energy metabolism is critical to feed efficiency and health of growing pigs. As mitochondria provide the intestine with the vast majority of ATP needed for proper functioning, disruptions to their activity are important with regard to energy generation. To understand intestinal mitochondrial function during *B. hyodysenteriae* challenge, live mitochondria were isolated from freshly excised ileum and colon tissue *via* differential centrifugation. Tissues were collected, placed in aerated bottles containing Krebs buffer, and transported on ice to the laboratory within 1 to 1.5 h of euthanasia. The serosal layer was removed, then approximately 10 g of tissue was weighed out, diced, and gently homogenized in 35 ml of mitochondrial isolation buffer [220 mM mannitol, 70 mM sucrose, 2 mM 4-(2-hydroxyethyl)-1-piperazineethanesulfonic acid (HEPES), 1 mM ethylene glycol-bis(β-aminoethyl ether)-N,N,N′,N′-tetraacetic acid (EGTA), and 0.5 mg/ml fatty acid-free bovine serum albumin (BSA), pH 7.4]. Homogenates were centrifuged (twice at 600 × g for 10 min at 4°C), the supernatant was strained through cheesecloth after each centrifugation, and the pellet containing cellular debris was discarded. Mitochondria were then pelleted by centrifugation 7,750 × g for 20 min at 4°C. The supernatant was decanted, and pellets were washed three times with 10 ml mitochondrial wash buffer (220 mM mannitol, 70 mM sucrose, 2 mM HEPES, and 0.5 mg/ml fatty acid-free BSA, pH 7.4). Washed mitochondria were resuspended in 3 ml mitochondrial wash buffer, and mitochondrial protein concentrations were determined *via* bicinchoninic acid (BCA) assay (Thermo Fisher Scientific, Waltham, MA). Mitochondria were diluted to a protein concentration of 2 mg/ml with mitochondrial wash buffer and stored at 4°C until use.

Mitochondrial reactive oxygen species (ROS) production was determined in isolated mitochondria using a 2′,7′-dichlorofluorescin diacetate (DCFH) assay described previously (20, 21). Fluorescence of DCFH was detected at an excitation/emission wavelength of 480/530 nm using a Cytation Hybrid Multi-Mode Reader using Gen 5 software (BioTek Instruments Inc., Winooski, VT). Mitochondrial hydrogen peroxide production was calculated from a hydrogen peroxide standard curve based on changes in relative fluorescence of DCFH. Samples were plated in triplicate using a black 96-well plate. Twenty units of superoxide dismutase (Sigma-Aldrich, St. Louis, MO) were added to each sample well to convert any superoxide produced into hydrogen peroxide. Either hydrogen peroxide standards or 90 μg of mitochondrial protein was added to each well after which 45 μl of an assay buffer (145 mM KCl, 30 mM HEPES, 5 mM KH_2_PO_4_, 3 mM MgCl_2_, 0.1 mM EGTA, 51 μM DCFH, 8 μM glutamate) was added. Plates were incubated at 37°C and read at 0, 5, 10, 15, and 20 min after adding the energy substrate. Readings were used to calculate the rate of hydrogen peroxide production per minute, expressed as mM hydrogen peroxide produced/min/mg mitochondrial protein.

Mitochondrial oxygen consumption was evaluated using a Seahorse XFe24 Extracellular Flux Analyzer (Seahorse Bioscience, North Billerica, MA) as previously described (22, 23). All reagents were made fresh daily from either new or frozen chemical stocks. Optimum concentrations of reagents to be injected were determined *via* preliminary titration trials. Mitochondria (40 μg) were plated in duplicate into a V7 XFe24 Tissue Culture Plate, and then diluted to 50 μl with mitochondrial assay buffer (220 mM mannitol, 70 mM sucrose, 5 mM KH_2_PO_4_, 5 mM MgCl_2_, 2 mM HEPES, 1 mM EGTA, 0.5 mg/ml BSA, pH 7.4). Blank wells contained 50 μl mitochondrial assay buffer. The plate was centrifuged at 2,000 × g for 10 min at 4°C to adhere mitochondria to the bottom of the plate. After centrifugation, 450 μl mitochondrial assay buffer + 5.5 mM glutamate + 5.5 mM malate (pH 7.4) were gently added to each well to provide mitochondria with a substrate. The plate was incubated in a non-CO_2_ incubator at 37°C for 8–10 min and then transferred to the XFe24 instrument for initiation of the experiment.

**Figure 1:**
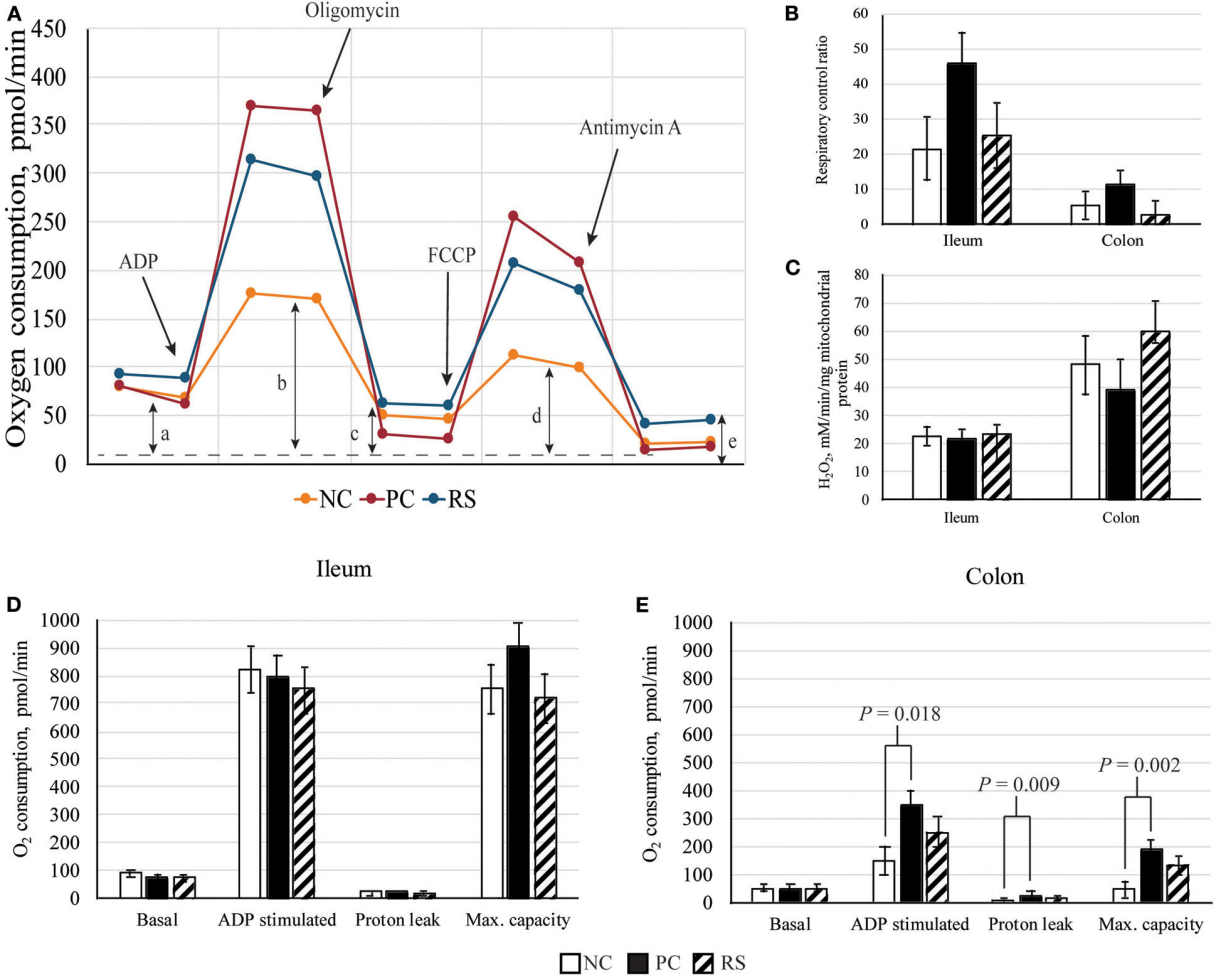
Mitochondrial parameters of sham-inoculated pigs (NC), *Brachyspira hyodysenteriae*-inoculated pigs (PC), and *Brachyspira hyodysenteriae*-inoculated pigs fed a diet containing 5% resistant starch and 5% sugar beet pulp (RS). **(A)** Schematic representation of graph obtained from Seahorse XFe24 Extracellular Flux analysis. Non-mitochondrial respiration (e) was subtracted from all values: (a) state 4, basal respiration; (b) state 3, ADP induced respiration; (c) state 4_o_, proton leak; and (d) state 3μ, FCCP induced maximal respiratory capacity (max. capacity). **(B)** Respiratory control ratios (State 3_μ_/4_o_) of ileal and colonic mitochondria. **(C)** Reactive oxygen species (ROS) production by isolated mitochondria. **(D,E)** Respiration of **(D)** ileal and **(E)** colonic mitochondria. Error bars represent lsmeans ± SEM, and *P*-values represent contrasts between either NC and PC or PC and RS pigs.

The XFe24 analyzer examines mitochondrial respiration under several states. First, mitochondria were measured in a coupled state with only substrate present (basal respiration). This was followed by the addition of 2 mM ADP (State 3; phosphorylating respiration), 2 μM oligomycin (State 4_o_; non-phosphorylating respiration, proton leak), 4 μM carbonyl cyanide 4-(trifluoromethoxy) phenylhydrazone (FCCP; State 3_u_; maximal uncoupler-stimulated respiration), and 4 μM antimycin A (loading control, blocks mitochondrial oxygen consumption). Efficient mitochondria should function by maintaining a low basal respiratory state and low proton leak, while being able to respond in the presence of ADP or FCCP to respire and consume oxygen quickly (22). A schematic of the assay is described in Figure 1A. From these measurements, respiratory control ratios (RCRs; State 3_u_/State 4_o_) were calculated, of which healthy mitochondria should have a high respiratory control (22). Mitochondrial respiration data were expressed as pmole O_2_ consumed per min.

### Dietary Analysis and Apparent Total Tract Digestibility

A representative feed sample from each complete diet was obtained for analysis. Fecal samples were collected for 3 consecutive days prior to each pig's individual necropsy date (dpi 10–16). Thus, fecal samples were collected to match similar stages of disease progression for all pigs. All freshly collected fecal samples were stored at −20°C until proximate analysis was conducted. For proximate analysis, fecal samples were thawed and homogenized within pig and then dried in a mechanical convection oven at 100°C.

Feed samples were ground through a 1-mm screen (Model ZM1; Retsch Inc., Newton, PA), and fecal samples were ground with a mortar and pestle prior to analysis. Proximate analysis was performed on feed and feces as previously described to determine apparent total tract digestibility (24). Dietary acid detergent fiber, neutral detergent fiber, insoluble fiber, and total dietary fiber analyses were conducted by the University of Missouri Experimental Station Chemical Laboratories (Columbia, MO). Dietary resistant starch and total starch concentrations were determined using the Megazyme Resistant Starch Assay Kit according to the manufacturer's instructions (Megazyme Ltd., Bray, Ireland).

All dietary and fecal samples were analyzed for dry matter (DM; AOAC method 930.15), titanium dioxide as described by Leone (25), nitrogen (**N**) using TruMac N (Leco Corporation, St. Joseph, MO), and gross energy (GE) using bomb calorimetry (Oxygen Bomb Calorimetry 6200; Parr Instruments, Moline, IL). Organic matter (OM) was determined using the ashing method and calculated as previously described (26). For each of the three treatments, apparent total tract digestibility (ATTD) coefficients for DM, OM, N, and GE were calculated using the index method (27).

### Ileal, Cecal, and Colonic Volatile Fatty Acid Concentrations

Ileal, cecal, and colonic volatile fatty acid (VFA) concentrations were determined as previously described (28). Briefly, contents were thawed for 1 h in a water bath at 55°C and then mixed thoroughly. Approximately 1.0 g of cecal and colonic digesta or 2.0 g of ileal digesta from each pig were weighed into a 15-ml polypropylene centrifuge tube. Cecal and colonic digesta were then diluted with 5 ml water and mixed overnight on a digital rocker. All samples were then centrifuged at 20,000 × g for 20 min at 4°C. For ileal samples, approximately 1 ml of supernatant was removed and placed in a new 15-ml polypropylene tube, to which 100 μl *o*-phosphoric acid was added to achieve a pH of 2.0–2.5. Ileal samples were then centrifuged at 4,000 × g for 10 min at 4°C. Exactly 1 ml of this supernatant was transferred to a 20-ml gas chromatography vial containing 0.3 g of NaCl. For cecal and colonic samples, after centrifugation at 20,000 × g, exactly 1 ml supernatant was removed and transferred to a 20-ml gas chromatography vial containing 0.3 g NaCl, to which 100 μl *o*-phosphoric acid was added to achieve a pH of 2.0–2.5. Samples were then analyzed *via* gas chromatography (Agilent 7890A Gas Chromatograph, Agilent Technologies Inc., Wilmington, DE). Samples were analyzed in duplicate, and values were multiplied to adjust for dilution. Concentrations are expressed as mM VFA per gram wet digesta. Furthermore, molar proportions (%) of the top 3 VFAs (acetate, propionate, butyrate) were calculated as the individual VFA/total VFA concentration × 100.

### Statistical Analysis

Statistical analysis of all data was performed in SAS 9.4 (SAS Institute, Cary, NC). The following mixed model was fitted to quantitative parameters:

$$\begin{array}{l} {\text{Y}}_{ij}=\mu +{\text{Trt}}_{i}+{\text{e}}_{ij} \end{array}$$

wherein Y_ij_ = the phenotype measured on animal *j*; Trt_i_ = effect of treatment (fixed effect; NC, PC, RS); and e_ij_ = error term of animal *j* subjected to treatment *i*, e_ij_ ~ N(0, $\sigma_{\text{e}}^{2}$). Least square means were determined using the LS means statement, and data are presented as least squares means with a pooled standard error. Contrast statements were used to determine the effect of either *B. hyodysenteriae* (NC vs. PC) or dietary treatment (PC vs. RS). Fisher's exact tests in the FREQ procedure were used to assess if treatment contributed to the number of pigs positive for *B. hyodysenteriae via* colon swab culture at necropsy or the number of pigs that had clinical SD at any point during the study. Log rank tests in the LIFETEST procedure were used to determine if diet contributed to the number of days it took for PC and RS pigs to develop clinical SD. For all analyses, differences were considered significant when *P* < 0.05 and a tendency when 0.05 ≤ *P* ≤ 0.10.

## Results

### Confirmation of Disease and Growth Performance

Colon swabs collected from all pigs at necropsy and selectively cultured for *B. hyodysenteriae* confirmed that sham-inoculated pigs were culture negative and that the *B. hyodysenteriae*-inoculated PC and RS pigs were all culture positive at the time of necropsy (Table 2). Soft to semisolid feces began to appear at dpi 6, and clinical SD (watery feces containing blood and mucus) was first observed at dpi 7 and 9 for PC and RS pigs, respectively. The timing of onset of clinical SD differed due to dietary treatment, with RS pigs tending to have a delayed onset (dpi 9) of clinical SD compared with dpi 7 for PC pigs (*P* = 0.083; Table 2). Overall, 100% of PC pigs and 75% of RS pigs developed clinical SD. The three RS pigs that had not developed SD at the end of the study (dpi 16) had soft to semisolid feces at necropsy, suggesting the possibility either of a mild form of disease or that clinical SD might manifest in several days' time.

**Table 2:** Confirmation of infection and clinical disease incidence of sham-inoculated pigs (NC), Brachyspira hyodysenteriae-inoculated pigs (PC), and *Brachyspira hyodysenteriae*-inoculated pigs fed a diet containing 5% resistant starch and 5% sugar beet pulp in replacement of 10% DDGS (RS).

	**Treatment**			***P*****-Values**	
	**NC**	**PC**	**RS**	**NC vs. PC**	**PC vs. RS**
Colon culture positive at necropsy^a^	0/12	12/12	12/12	<0.001	1.000
Observed clinical disease^a^	0/12	12/12	9/12	<0.001	0.217
Median days to clinical disease	–	10	12	–	0.083

a *Number of pigs positive for the observed phenotype out of the total number of pigs in the treatment group*.

Pig performance was recorded in the 14 days prior to challenge. In this pre-challenge period, average daily gains differed, as RS pigs had greater ADG than PC pigs (*P* < 0.004; Table 3). Performance differences between NC and PC pigs were not observed in the pre-challenge period. Average daily feed intake during this time did not differ, but RS pigs had greater G:F than PC pigs (*P* = 0.023). However, despite differences in growth during the pre-challenge period, BW at dpi 0 did not differ between treatments (*P* ≥ 0.360; Table 3).

**Table 3:** Average daily gain (ADG), average daily feed intake (ADFI), and feed efficiency (Gain:Feed) of sham-inoculated pigs (NC), *Brachyspira hyodysenteriae*-inoculated pigs (PC), and *Brachyspira hyodysenteriae*-inoculated pigs fed a diet containing 5% resistant starch and 5% sugar beet pulp in replacement of 10% DDGS (RS).

	**Treatment**			***P*****-Values**		
	**NC**	**PC**	**RS**	**SEM**	**NC vs. PC**	**PC vs. RS**
**Pre-challenge (dpi−14 to 0)**						
ADG, kg/d	0.76	0.81	0.99	0.042	0.392	0.004
ADFI, kg/d	1.53	1.48	1.67	0.089	0.700	0.151
Gain:Feed	0.50	0.53	0.60	0.018	0.250	0.023
**dpi 0–4**						
ADG, kg/d	1.33	1.22	1.36	0.077	0.351	0.223
ADFI, kg/d	1.80	2.02	1.70	0.102	0.459	0.024
Gain:Feed	0.82	0.71	0.68	0.034	0.105	0.351
**dpi 4–8**						
ADG, kg/d	0.89	0.13	0.78	0.170	0.003	0.011
ADFI, kg/d	1.98	1.54	1.96	0.118	0.014	0.018
Gain:Feed	0.46	0.01	0.38	0.111	0.006	0.022
**dpi 8–12**						
ADG, kg/d	1.01	−0.56	−0.52	0.293	<0.001	0.916
ADFI, kg/d	2.09	1.03	1.05	0.231	0.002	0.963
Gain:Feed	0.49	−1.33	−0.83	0.439	0.008	0.590
**dpi 12 to necropsy**						
ADG, kg/d	0.68	−0.65	−0.51	0.238	<0.001	0.634
ADFI, kg/d	2.41	0.74	0.67	0.293	<0.001	0.856
Gain:Feed	0.28	−2.94	−2.44	1.365	0.084	0.786
**Overall (dpi 0 to necropsy)**						
ADG, kg/d	1.03	0.16	0.31	0.070	<0.001	0.154
ADFI, kg/d	1.96	1.22	1.59	0.098	<0.001	0.011
Gain:Feed	0.54	0.07	0.19	0.040	<0.001	0.052
dpi 0 BW, kg	34.8	36.8	37.0	1.495	0.360	0.904
Necropsy BW, kg	49.5	37.5	43.5	2.192	0.001	0.065

After *B. hyodysenteriae* inoculation, growth, feed intake, and feed efficiency were all negatively impacted in *B. hyodysenteriae*-inoculated pigs (Table 3). In the first 4 dpi, ADG and G:F did not differ, but ADFI was reduced in RS pigs compared with PC pigs (*P* = 0.024). From dpi 4 to 8, ADG was reduced in PC pigs compared with NC and RS pigs 85 and 83%, respectively (*P* < 0.05 for both comparisons). Feed intake and G:F were also reduced in PC pigs compared with NC pigs in this period (*P* < 0.05 for both analyses). From dpi 8 to 12, ADG, ADFI, and G:F were all reduced in PC pigs when compared with NC pigs (*P* ≤ 0.008 for all analyses), while PC and RS pigs did not differ from each other. Similarly, from dpi 12 until the end of the study, ADG and ADFI were reduced in PC pigs compared with NC pigs (*P* < 0.001 for both analyses) and did not differ between PC and RS pigs. However, G:F only tended to be reduced in PC pigs compared with NC pigs (*P* = 0.084).

Overall, ADG in the post-challenge period was reduced in both PC and RS pigs compared with NC pigs (*P* < 0.001; Table 3), and ADG did not differ between PC and RS pigs. Average daily feed intake was reduced 38% in PC pigs compared with NC pigs (*P* < 0.001) and was reduced 23% in PC pigs when compared with RS pigs (*P* = 0.011). Overall, G:F was lower in PC pigs compared with NC pigs (*P* < 0.001) and tended to be lower in PC pigs compared with RS pigs (*P* = 0.052).

### *Ex vivo* Intestinal Integrity and Nutrient Transport

*Ex vivo* markers of intestinal integrity and active nutrient transport at necropsy are presented in Table 4. In the ileum, intestinal integrity markers of transepithelial resistance and FD4 macromolecule permeability did not differ (*P* > 0.10). Active transport of glucose tended to be greater in PC pigs compared with NC pigs (*P* = 0.060), while active transport of glutamine did not differ (*P* > 0.10). In the colon, transepithelial resistance was increased in PC pigs compared with NC pigs (*P* = 0.001) and was increased in PC pigs compared with RS pigs (*P* = 0.045). Further, colon FD4 permeability was reduced in PC pigs compared with NC pigs (*P* = 0.025) and did not differ between PC and RS pigs.

**Table 4:** *Ex vivo* intestinal integrity and function parameters of sham-inoculated pigs (NC), *Brachyspira hyodysenteriae*-inoculated pigs (PC), and *Brachyspira hyodysenteriae-*inoculated pigs fed a diet containing 5% resistant starch and 5% sugar beet pulp (RS).

	**Treatment**			***P*****-Values**		
	**NC**	**PC**	**RS**	**SEM**	**NC vs. PC**	**PC vs. RS**
**Ileum**						
Transepithelial resistance^a^	33.9	38.8	33.7	3.39	0.423	0.239
Glucose transport, μA^b^	35.6	63.5	57.5	10.5	0.060	0.676
Glutamine transport, μA^b^	8.5	12.4	14.6	2.21	0.206	0.467
FD4 permeability^c^	164	209	136	26.4	0.234	0.059
**Colon**						
Transepithelial resistance^a^	39.1	53.1	45.0	2.75	0.001	0.045
FD4 permeability^c^	187	92.2	75.8	24.8	0.010	0.629

a *Ω × cm^2^*.

b *Active absorption calculated by subtracting μA before substrate (glucose or glutamine) from μA after substrate addition*.

c *Apparent permeability coefficient for macromolecule [fluorescein isothiocyanate-dextran 4 kDa (FD4)] permeability*.

### Mitochondrial Function

Mitochondrial respiration measurements and mitochondrial ROS production are presented in Figure 1. In the ileum, basal respiration, ADP-stimulated respiration, oligomycin-induced proton leak, and FCCP-induced maximal capacity were not different among treatments (*P* > 0.10; Figure 1D); however, respiratory control ratios tended to be increased in PC pigs compared with RS pigs (*P* = 0.086; Figure 1B). Ileal mitochondrial ROS production did not differ (*P* > 0.10; Figure 1C).

In the colon, basal respiration did not differ. However, ADP-stimulated respiration (*P* = 0.018), proton leak (*P* = 0.009), and FCCP-induced maximal capacity (*P* = 0.002) were all elevated in PC pigs compared with NC pigs (Figure 1E). For these measurements, PC and RS pigs did not differ from one another. Mitochondrial respiratory control ratios and mitochondrial ROS production did not differ in the colon (*P* < 0.10 for all contrasts).

### Digesta pH and Volatile Fatty Acid Concentrations

In the ileum, there were no differences in digesta pH (*P* ≥ 0.223; Table 5). Furthermore, there were no differences in total VFA concentrations in the ileum (*P* ≥ 0.579; Table 5) or in individual concentrations of any of the VFAs, except for butyrate and isocaproate. Butyrate concentrations were greater in NC pigs compared with PC pigs (*P* = 0.045) and did not differ between PC and RS pigs. Isocaproate concentrations were greater in NC pigs compared with PC pigs (*P* = 0.033) and did not differ between PC and RS pigs. Molar proportions (%) of acetate and propionate did not differ in the ileum, but the molar proportion of butyrate was greater in PC pigs compared with NC pigs (*P* = 0.032) and did not differ between PC and RS pigs.

**Table 5:** Ileum digesta pH, volatile fatty acid (VFA) concentrations, and molar proportions of sham-inoculated pigs (NC), *Brachyspira hyodysenteriae*-inoculated pigs (PC), and *Brachyspira hyodysenteriae*-inoculated pigs fed a diet containing 5% resistant starch and 5% sugar beet pulp (RS).

	**Treatment**				***P*****-Values**	
	**NC**	**PC**	**RS**	**SEM**	**NC vs. PC**	**PC vs. RS**
pH	6.95	7.16	7.27	0.125	0.223	0.517
**Concentration, mM/g wet digesta**						
Acetate	14.93	16.00	19.54	3.575	0.827	0.489
Propionate	0.089	0.146	0.205	0.039	0.300	0.292
Butyrate	1.143	0.598	0.760	0.193	0.045	0.546
Isobutyrate	0.033	0.033	0.036	0.012	0.975	0.836
Valerate	0.0008	0.0013	0.0016	0.0004	0.303	0.613
Isovalerate	0.094	0.068	0.068	0.026	0.467	0.992
Caproate	0.0020	0.0014	0.0015	0.0003	0.252	0.957
Isocaproate	0.0031	0.0021	0.0017	0.0003	0.033	0.382
Heptanoate	0.0004	0.0004	0.0002	0.0001	0.965	0.324
Total	16.36	16.20	19.29	3.896	0.976	0.579
**Molar proportion, %**						
Acetate	92.21	94.36	94.80	0.951	0.105	0.743
Propionate	0.620	0.919	0.893	0.140	0.134	0.899
Butyrate	6.20	3.87	3.57	0.768	0.032	0.783

In the cecum, pH values were higher in PC pigs compared with NC pigs (*P* < 0.001; Table 6) and did not differ between PC and RS pigs. Total and individual VFA concentrations were also different among treatments. Concentrations of acetate, propionate, butyrate, and total VFA concentrations were all lesser in PC pigs compared with NC pigs (*P* < 0.05 for all analyses). Conversely, concentrations of isobutyrate, valerate, isovalerate, and heptanoate were greater in both PC pigs compared with NC pigs (*P* < 0.05 for all analyses). Concentrations of isobutyrate and isovalerate tended to be greater in PC pigs compared with RS pigs (*P* = 0.067 and *P* = 0.057, respectively). Concentrations of caproate and isocaproate tended to be greater in PC pigs compared with NC pigs (*P* = 0.055 for both), and concentrations of caproate were greater in PC pigs compared with RS pigs (*P* = 0.031). Molar proportions of VFAs in the cecum did not differ among treatments.

**Table 6:** Cecum digesta pH, volatile fatty acid (VFA) concentrations, and molar proportions of sham-inoculated pigs (NC), *Brachyspira hyodysenteriae*-inoculated pigs (PC), and *Brachyspira hyodysenteriae*-inoculated pigs fed a diet containing 5% resistant starch and 5% sugar beet pulp (RS).

	**Treatment**				***P*****-Values**	
	**NC**	**PC**	**RS**	**SEM**	**NC vs. PC**	**PC vs. RS**
pH	5.61	6.37	6.20	0.100	<0.001	0.223
**Concentration, mM/g wet digesta**						
Acetate	83.51	59.08	59.88	7.893	0.030	0.942
Propionate	24.01	17.61	16.92	1.801	0.014	0.785
Butyrate	9.403	6.157	6.945	0.799	0.005	0.481
Isobutyrate	0.223	1.403	0.944	0.175	<0.001	0.067
Valerate	1.166	1.976	1.578	0.202	0.007	0.173
Isovalerate	0.215	1.809	1.171	0.234	<0.001	0.057
Caproate	0.055	0.157	0.198	0.037	0.055	0.423
Isocaproate	0.016	0.021	0.016	0.002	0.055	0.031
Heptanoate	0.004	0.018	0.018	0.004	0.014	0.936
Total	118.6	87.89	87.75	9.762	0.027	0.992
**Molar proportion, %**						
Acetate	69.12	66.96	68.02	1.820	0.397	0.677
Propionate	20.89	20.07	19.63	0.955	0.541	0.739
Butyrate	8.49	6.89	7.83	0.713	0.116	0.349

In the colon, pH values were higher in PC pigs compared with NC pigs (*P* = 0.007; Table 7) and did not differ between PC and RS pigs. Concentrations of acetate tended to be greater in the colon digesta of NC pigs compared with PC pigs (*P* = 0.053; Table 7). Concentrations of isobutyrate, isovalerate, and isocaproate were increased in PC pigs compared with NC pigs (*P* < 0.050 for all analyses). Concentrations of caproate were lower in PC pigs compared with NC pigs (*P* = 0.043). Total VFA concentrations were reduced in PC pigs compared with NC pigs (*P* = 0.019). Molar proportions of acetate and propionate did not differ among treatments; however, molar proportions of butyrate were reduced in PC pigs compared with NC pigs (*P* = 0.011).

**Table 7:** Colon digesta pH, volatile fatty acid (VFA) concentrations, and molar proportions of sham-inoculated pigs (NC), *Brachyspira hyodysenteriae*-inoculated pigs (PC), and *Brachyspira hyodysenteriae*-inoculated pigs fed a diet containing 5% resistant starch and 5% sugar beet pulp (RS).

	**Treatment**				***P*****-Values**	
	**NC**	**PC**	**RS**	**SEM**	**NC vs. PC**	**PC vs. RS**
pH	6.43	6.71	6.75	0.069	0.007	0.652
**Concentration, mM/g wet digesta**						
Acetate	61.64	51.03	48.41	3.809	0.053	0.639
Propionate	18.07	16.20	16.73	1.495	0.383	0.810
Butyrate	5.747	4.970	6.276	0.546	0.291	0.066
Isobutyrate	0.541	1.245	1.710	0.154	0.002	0.036
Valerate	1.349	1.500	1.818	0.146	0.469	0.141
Isovalerate	0.667	1.735	2.234	0.225	0.002	0.126
Caproate	0.203	0.092	0.118	0.032	0.043	0.496
Isocaproate	0.015	0.022	0.022	0.001	0.002	0.780
Heptanoate	0.019	0.020	0.020	0.006	0.906	0.955
Total	90.38	71.60	76.83	5.572	0.019	0.512
**Molar proportion, %**						
Acetate	68.64	66.77	64.16	1.516	0.390	0.232
Propionate	19.85	20.30	20.50	0.689	0.642	0.838
Butyrate	8.47	6.29	7.91	0.575	0.011	0.054

### Apparent Total Tract Digestibility

**Figure 2:**
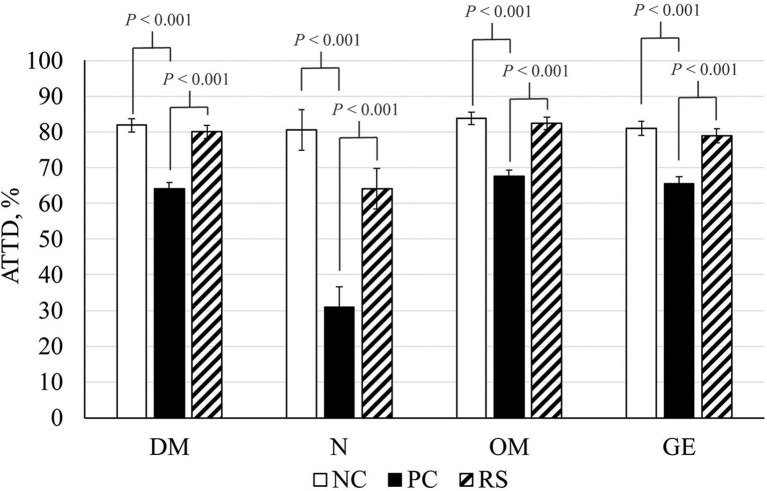
Apparent total tract digestibility (ATTD) coefficients of dry matter (DM), nitrogen (N), organic matter (OM), and gross energy (GE) of sham-inoculated pigs (NC), *Brachyspira hyodysenteriae*-inoculated pigs (PC), and *Brachyspira hyodysenteriae*-inoculated pigs fed a diet containing 5% resistant starch and 5% sugar beet pulp (RS). Error bars represent lsmeans ± SEM, and *P*-values represent contrasts between either NC and PC or PC and RS pigs.

Reductions in DM, N, OM, and GE ATTD coefficients were observed in response to *B. hyodysenteriae* inoculation. Reductions of 20, 72, 22, and 24% were observed in DM, N, OM, and GE digestibility, respectively, in PC pigs compared with NC pigs (*P* < 0.001 for all analyses; Figure 2). Interestingly, ATTD coefficients of DM, N, OM, and GE were greater in RS pigs compared with PC pigs (*P* < 0.001 for all analyses).

## Discussion

In the post-nursery period, pigs clinically affected with SD become anorexic and dehydrated, antagonizing growth and health (2). SD also compromises the health of the intestinal epithelium, inducing mucosal necrosis, neutrophil infiltration, and hemorrhage (29). Intestinal epithelial health comprises an array of physiological and functional elements, including barrier permeability, nutrient digestion and absorption, a stable microbiome, host metabolism and energy generation, mucus layer development, and mucosal immune responses (30). Disruptions to these elements during stress events such as during SD result in intestinal dysbiosis, reducing animal well-being, growth, and feed efficiency. Our group has previously demonstrated that there are differences in how gastrointestinal physiology is affected under various bacterial (31) and viral (32–34) pathogen challenges, highlighting the importance of characterizing the differential changes to physiology that may occur during different disease states. However, the extent to which *B. hyodysenteriae* and its associated disease directly modulates intestinal function and integrity in grower pigs, and how these relate to reduced performance, has been poorly defined. Further, dietary fiber is known to alter SD development and severity; however, the physiological mechanisms underlying these phenomena are unclear. Thus, the objectives of this study were to (1) evaluate intestinal barrier permeability, digestive function, and metabolism during a *B. hyodysenteriae* challenge and (2) determine if replacement of dietary insoluble fiber with highly fermentable fiber sources would mitigate SD by improving parameters of intestinal health.

Growth performance of pigs during the development and onset of SD is variable and has been poorly characterized due to factors such as pig age, study duration, disease severity, and researchers failing to include naive controls or failing to report growth performance entirely. In general, *B. hyodysenteriae* challenge has been associated with anywhere from a 25 to 75% reduction in ADG compared with healthy control pigs (13, 35, 36), reductions which may or may not be mitigated by dietary factors. Wilberts et al. (13) found that pigs inoculated with *B. hyodysenteriae* had reductions in growth regardless of whether they were fed a control diet or fed an SD-exacerbating diet containing high levels of lowly fermentable insoluble fiber. Other researchers have reported reductions in ADG to be mitigated with supplementation of conjugated linoleic acid (35). Further, several researchers have documented that pigs fed highly fermentable inulin grow faster than those fed either a control diet or a diet high in lowly fermentable fibers following *B. hyodysenteriae* challenge (12, 36). Herein, we reported reductions in growth to be significantly reduced as early as 8 dpi and continuing until the end of the study. Highly fermentable fiber delayed the reductions observed in ADG; however, these pigs still had dramatic reductions in growth. In fact, by dpi 12, pigs in both *B. hyodysenteriae*-challenged groups were losing BW. Much of this is likely attributed to a loss of water due to dehydration, as 87% of *B. hyodysenteriae*-challenged pigs developed watery, bloody, and mucoid feces prior to the end of the study. In general, diarrheic pigs lose body water and non-aqueous tissue mass in an 80/20 ratio, thus ~20% of this dramatic decline in BWs can be attributed to losses in tissue mass (37). Although the pigs herein were not given time to recover, it is likely that these dramatic losses in BW during peak infection would not be fully recompensated by the pig and would increase time to market BW. Further, growth performance and feed efficiency reductions support alterations to homeostasis and a basis to evaluate disruptions to intestinal health.

One key aspect of intestinal health and function is barrier permeability, primarily regulated by the tight junction complexes forming a seal between epithelial cells (38). Increased barrier permeability is documented during enteric challenges such as porcine epidemic diarrhea virus (33) and increases the pig's susceptibility to further challenge by luminal toxins or bacteria. To the authors' knowledge, this is the first published report examining ileum permeability in *B. hyodysenteriae*-challenged pigs. Although *B. hyodysenteriae* is a large intestinal pathogen and does not directly impact the small intestinal epithelium, reductions in feed intake alone can increase barrier permeability and active nutrient transport (18, 39). Further, Kaleczyc et al. (40) found increases in ileal tissue concentrations of certain neuropeptides and changes to percentages of some lymphocyte populations in ileal Peyer's patches of pigs with clinical SD, highlighting neuro-immune cross-talk between intestinal segments during SD. However, we observed very few changes in ileal *ex vivo* integrity, active nutrient transport, or mitochondrial metabolism. Additionally, limited changes to ileal VFA concentrations and proportions were observed. Limited alterations to ileal physiology are supported by observations by Argenzio (41), who demonstrated that pigs in the early stages of SD do not have any changes to small intestinal absorption of glucose or fluid. Additionally, Schweer et al. (6) observed no reduction in apparent ileal digestibility coefficients of DM, N, total amino acids, or GE despite reductions in apparent total tract digestibility of these same parameters, suggesting ileal digestive function is unhindered. This suggests that despite severe colonic disease, ileal function and integrity remain largely unchanged during SD.

*B. hyodysenteriae* induces neutrophilic inflammation in the colon (42), which may lead to internalization of tight junction proteins and increased barrier permeability (43), rendering the pig susceptible to further insult from other pathogens or toxins. However, previous researchers have found no reduction in colon transepithelial resistance (8) and no difference in macromolecule (mannitol or polyethylene glycol-400) permeability (5) in pigs with SD. In agreement with these reports, we did not see any reduction in colon integrity; in fact, transepithelial resistance was increased and FD4 permeability decreased in pigs challenged with *B. hyodysenteriae*. Although a necrotic section of tissue was not specifically selected for these permeability measures, macroscopic lesions and inflammation were widely present around the apex of the spiral colon where explants were removed, thus the explant chosen was representative of a lesioned area. Regardless, the reductions in *ex vivo* permeability observed can likely be attributed to a thickened mucus layer, as mucus hypersecretion is a classic response to *B. hyodysenteriae* infection (4). Mucin production increases 5-fold and the mucus layer is visibly thicker in experimentally infected pigs compared with healthy controls (4). A thicker mucus layer would cause both an elevation in electrical resistance and a reduction in permeability to macromolecules (44), as what we observed. However, during SD, the mucus layer loses its parallel striations and becomes disorganized, so it may be unstable and less beneficial to the pig in preventing bacteria from reaching the epithelium (4). Additionally, these permeability measures, when skewed by mucus thickness, do not indicate if tight junction complex integrity itself is affected. Further work would be needed to determine if this is the case.

In addition to forming a selectively permeable seal against the luminal environment, the intestinal epithelium is also responsible for digestion and absorption of water and dietary nutrients. Reductions in digestive function reduce nutrients available for growth and lean accretion. To evaluate digestive function during SD, ATTD of nitrogen and energy was evaluated. Herein, PC pigs had reductions in DM, N, OM, and GE ATTD coefficients of 20, 72, 22, and 24%, respectively, compared with NC pigs. The incredibly low nitrogen ATTD observed in PC pigs is likely attributed to excessive mucus secretion, which would increase endogenous losses of N, thus decreasing digestibility. In support of this, increased hindgut N appearance is observed in *B. hyodysenteriae-*infected pigs (6). Reductions in all ATTD coefficients are consistent with previous reports in *B. hyodysenteriae*-infected pigs by Schweer et al. (6), although far more dramatic. However, fecal samples from Schweer et al. (6) were collected at the same dpi (9–11) for all pigs, whereas fecal samples from the current study were collected immediately prior to each pig's individual necropsy date, thus ensuring nearly all pigs were clinically afflicted at the time of collection. Further, differences in clinical severity may also explain the greater reductions in the current experiment. Diarrhea reduces passage time through the gut, leading to poor breakdown of feedstuffs and limiting absorption (45, 46). As such, greater severity of diarrhea would lead to greater reductions in ATTD coefficients and would exacerbate the attenuated growth already occurring due to reduced feed intake. Interestingly, ATTD coefficients were greater in RS pigs compared with PC pigs. This suggests that the highly fermentable diet was able to prevent *B. hyodysenteriae*-induced digestive dysfunction, which could mitigate performance losses in a less severe challenge.

Maintaining a stable microbiota is critical to ensure pig well-being. Not only do commensal microbes prevent against pathogenic bacterial colonization, their metabolic end products can be used by the pig for energy generation. VFAs, the endpoints of microbial fermentation, are often used as a marker of microbial metabolism (47). The most widely produced VFAs include acetate, propionate, and butyrate. Of these, butyrate has been studied the most extensively with regards to its impact on colon metabolism (48). Health status is known to alter microbial populations, and thus their metabolic end products. Nursery pigs challenged with an F18 *E. coli* had reduced total VFA concentrations when compared with healthy controls (49). Similarly, in the current experiment total concentrations of VFAs were reduced in both the cecum and colon of pigs challenged with *B. hyodysenteriae*, accompanied by an increase in luminal pH. An increase in pH and reduction in VFA concentrations is not surprising, considering pigs had watery, malabsorptive diarrhea, and thus diluted digestive contents. Further, reduced feed intake would lower available substrates, reducing microbial fermentation and thus products of their metabolism. While concentrations of many VFAs were reduced in the cecum and colon, an increase in concentrations of the iso-acids, or branched-chain fatty acids, was observed in *B. hyodysenteriae*-challenged pigs. Branched-chain fatty acids are produced exclusively *via* branched-chain amino acid metabolism (50), indicating increased protein fermentation in *B. hyodysenteriae*-challenged pigs. This could be due to fermentation of mucus, which is of abundance in clinically affected pigs, or increased dietary protein reaching the large intestine due to reduced transit time in the ileum. In addition to branched-chain fatty acids, other products of protein fermentation include ammonia, hydrogen sulfide, *p*-cresol, and phenols, which may have toxic effects on the colonic epithelium (51).

The intestine must be able to maintain barrier integrity and perform nutrient absorption with minimal energy usage, so efficient metabolism is critical for intestinal health. Colonocytes preferentially use butyrate as fuel, sparing glucose for the rest of the body (52). In the healthy colon, butyrate is metabolized *via* mitochondrial β-oxidation and energy generated through oxidative phosphorylation, maintaining epithelial hypoxia (7). Toxic by-products, such as those produced through protein fermentation (including ammonia, hydrogen sulfide, *p*-cresol, and phenols), or stress events may alter these processes (53). Particularly, hydrogen sulfide is known to inhibit cytochrome c oxidase, the terminal oxidase of the mitochondrial electron transport chain, which catalyzes reduction of dioxygen to water and harnesses the free energy of that reaction to catalyze phosphorylation of ADP to ATP (53, 54). Human colonic HT-29 GLC–/+ cells treated with hydrogen sulfide had reduced maximal respiratory capacity and increased oxygen consumption in the presence of oligomycin, suggesting mitochondrial inefficiency and increased proton leak (55). However, in the current study, PC pigs had increased mitochondrial maximal respiratory capacity, suggesting that energy generation may be more efficient in PC pigs, potentially as an adaptive mechanism or due to infiltrating immune cells, which are highly metabolic. However, PC pigs also had increased oxygen consumption in the presence of F_0_/F_1_ ATPase inhibitor oligomycin, indicating enhanced proton leak (55). Although this may be a by-product of overall greater mitochondrial metabolic rates, excessive proton leak leads to the formation of ROS, which can damage the epithelium. Piglets challenged with lipopolysaccharide (LPS) had increased jejunal mitochondrial ROS production, which was associated with increased mitophagy, adding to intestinal dysbiosis and acting as a source of inefficiency (56). In the current experiment, mitochondrial ROS production was not different among treatments, suggesting the evidence of enhanced proton leak was not leading to oxidative stress and acting as a detriment to intestinal metabolism. Taken together, this *B. hyodysenteriae* challenge appeared to impact intestinal health *via* reducing digestibility of nutrients, reducing microbial metabolism and inducing mucus hypersecretion, which led to a shift toward protein fermentation in the large intestine. Thus, our next objective was to determine if dietary manipulation could mitigate SD *via* improving these parameters of intestinal health.

Dietary manipulation has been explored extensively as a method to prevent SD. However, there is much discord in the literature with regard to which diet components are beneficial or detrimental when pigs are at risk for *B. hyodysenteriae* infection. Addition of 30% corn DDGS to diets based on corn and soybean meal leads to pigs shedding bacteria and developing SD sooner than those without DDGS, suggesting that increasing insoluble fibers may be detrimental with regard to developing SD (13). Additionally, several studies have found that feeding pigs highly digestible diets based on cooked rice (14, 57) or cornstarch (6) reduces the incidence of SD upon experimental challenge. Cooked white rice diets were also associated with increased digesta pH and reduced VFA concentrations, suggesting that limiting microbial fermentation in the large intestine hinders *B. hyodysenteriae* colonization (14, 57). Adding a highly fermentable fiber to a cooked white rice diet resulted in greater disease incidence, further implicating microbial fermentation as a risk factor for developing SD (11). However, attempts to reproduce these results with cooked white rice by using parboiled rice did not reduce the incidence of SD (58, 59). The reason behind these differential reports may lie in rice processing, as cooked rice gelatinizes and is rapidly digested by the body, while parboiled rice retrogrades and acts as a resistant starch, resistant to small intestinal digestion and rapidly fermented by microbes in the large intestine (60).

In contrast to work by Pluske et al. (57) and Siba et al. (14), several researchers have found that dietary addition of fermentable carbohydrates to pig diets mitigates SD. Specifically, Thomsen et al. (36) found that a diet containing highly fermentable dried chicory roots (fructans) and lupins (galactans) completely protected pigs from SD upon experimental challenge. A series of experiments by Hansen et al. (12, 15) further examined the protective effects of fermentable carbohydrates in pigs fed triticale- and barley-based diets. This work demonstrated that diets containing the fermentable fiber source inulin, but not lupins, were able to prevent the development of SD. However, diets containing both inulin and lupins were able to delay disease onset and increase ADG during the study period (12). Furthermore, a high concentration of inulin (greater than 40–80 g/kg diet) was necessary to provide this protective effect (15). Interestingly, increased dietary inulin was associated with increased VFAs, suggesting an increase in microbial fermentation (15). However, this may be confounded with differences in disease severity, as pigs with lesser disease incidence would have greater digesta dry matter and thus greater VFA concentrations by default, as watery intestinal contents would dilute VFA concentrations (15).

Although differences in study design, base diet, pig age, adaptation period, etc., make it difficult to compare between these studies, some connections can be made. In general, in the case of diets based on highly digestible carbohydrates, increasing fibers, even those that are highly fermentable, increase disease (11, 57). Conversely, when diets are formulated from ingredients that are less highly digestible (barley, triticale), addition of fermentable fiber is beneficial (12, 15). In the case of US swine producers, feeding high levels of highly digestible ingredients such as cooked white rice is not common, as most diets are based on ground corn and soybean meal. Thus, the addition of highly fermentable fibers may prove beneficial. Indeed, in the current study, addition of fermentable fibers in the form of resistant starch and sugar beet pulp, while not protective, delayed the onset of clinical SD and reductions in ADG, as ADG was greater in RS pigs than in PC pigs from dpi 4 to 8. Pigs fed resistant starch also had greater ATTD coefficients than PC pigs, suggesting disease mitigation. However, the current study does not make it possible to evaluate how pigs on the RS diet may perform in the absence of *B. hyodysenteriae* challenge, thus disease mitigation and diet effects cannot be fully differentiated. Further, as nearly all pigs in the current study developed SD and were euthanized at peak infection, disease mitigation is difficult to determine.

Taken together, these data indicate that SD induced by *B. hyodysenteriae* does not appear to reduce *ex vivo* small intestinal integrity and in fact appears to increase *ex vivo* colon integrity likely due to the high production of epithelial surface mucus by the colon. Further, reductions in ATTD and changes to digesta pH and digesta VFA concentrations all support the pathogenesis of malabsorptive diarrhea and increased mucus production. Finally, replacement of insoluble fiber with highly fermentable fibers delayed the onset of and appeared to mitigate SD; however, a longitudinal study or study with lesser disease severity is needed to confirm these findings.

## Data Availability Statement

The raw data supporting the conclusions of this article will be made available by the authors, without undue reservation.

## Ethics Statement

The animal study was reviewed and approved by Iowa State University Institutional Animal Care and Use Committee.

## Author Contributions

All authors contributed to the design of the study, interpretation of the results, and assisted with manuscript editing. Performed live animal experiments: EH and SL. Performed laboratory experiments: EH. Wrote the manuscript: EH.

## Conflict of Interest

The authors declare that the research was conducted in the absence of any commercial or financial relationships that could be construed as a potential conflict of interest.
